# Assessment of Various Toxicity Endpoints in Duckweed (*Lemna minor*) at the Physiological, Biochemical, and Molecular Levels as a Measure of Diuron Stress

**DOI:** 10.3390/biology10070684

**Published:** 2021-07-20

**Authors:** Hojun Lee, Stephen Depuydt, Kisik Shin, Soyeon Choi, Geonhee Kim, Yun Haeng Lee, Joon Tae Park, Taejun Han, Jihae Park

**Affiliations:** 1 Department of Marine Science, Incheon National University, 119 Academy-ro, Yeonsu-gu, Incheon 22012, Korea; hojun.lee@ugent.be (H.L.); chlthdus0501@hanmail.net (S.C.); jkh-011@hanmail.net (G.K.); Taejun.Han@ghent.ac.kr (T.H.); 2 Ghent University Global Campus, 119-5 Songdomunhwa-ro, Yeonsu-gu, Incheon 21985, Korea; Stephen.Depuydt@ghent.ac.kr; 3 Water Environmental Engineering Research Division, National Institute of Environmental Research, 42 Hwangyeong-ro, Seo-gu, Incheon 22689, Korea; envi95@korea.kr; 4 Division of Life Sciences, Incheon National University, 119 Academy-ro, Yeonsu-gu, Incheon 22012, Korea; licdbsgod@inu.ac.kr (Y.H.L.); joontae.park@inu.ac.kr (J.T.P.)

**Keywords:** chlorophyll *a* fluorescence, diuron, *Lemna minor*, gene transcription, toxicity

## Abstract

**Simple Summary:**

The presence of diuron in a variety of environments has been reported worldwide to exert serious harm to human health and the ecosystem. HPLC and mass spectrometry are highly specific and sensitive methods for herbicide detection, but they have several drawbacks including complex sample preparation procedures, the need for expensive chemicals and equipment, and interference from secondary contaminants during analysis. In addition, these purely chemical approaches do not provide ecologically meaningful information on temporal changes in terms of exposure or the interactive effects of pollutants. In order to compensate for these limitations, biological assays have been used to assess pollutant-induced ecological risks. *Lemna minor* is an attractive experimental model organism that has been used for decades for the prospective risk assessment of pesticides. In the current study, we examined the effects of diuron on *L. minor* using different endpoints at the physiological (growth and photosynthetic efficiency), biochemical (pigment biosynthesis and reactive oxygen species (ROS) levels), and molecular (gene transcription) levels. Our findings provide important insight into the relative sensitivity of different endpoints for diuron toxicity assessment. In addition, they shed light on the toxicity mechanisms of diuron in a model aquatic macrophyte species.

**Abstract:**

The common, broad-spectrum herbicide diuron poses some risks to the environment due to its long persistence and high toxicity. Therefore, the effective monitoring of diuron residues will inform efforts to assess its impacts on ecosystems. In this study, we evaluated the toxicity targets of diuron in the model aquatic macrophyte *Lemna minor* at the physiological (growth and photosynthetic efficiency), biochemical (pigment biosynthesis and reactive oxygen species (ROS) levels), and molecular (*rbcL* transcript) levels. The toxicity of diuron was detectable after 48 h of exposure and the order of sensitivity of toxicity endpoints was gene transcription > maximum electron transport rate (ETR_max_) > non-photochemical quenching (NPQ) > maximum quantum yield (*F_v_/F_m_*) > ROS > fresh weight > chlorophyll *b* > chlorophyll *a* > total frond area > carotenoids. Under diuron stress, pigment, ROS, and gene transcript levels increased while frond area, fresh weight, and photosynthesis (*F_v_/F_m_* and ETR_max_) gradually decreased with the increasing duration of exposure. Notably, ROS levels, *F_v_/F_m_*, frond area, and fresh weight were highly correlated with diuron concentration. The growth endpoints (frond area and fresh weight) showed a strong negative correlation with ROS levels and a positive correlation with *F_v_/F_m_* and ETR_max_. These findings shed light on the relative sensitivity of different endpoints for the assessment of diuron toxicity.

## 1. Introduction

Herbicides have widespread applications in agriculture, horticulture, and the maintenance of green spaces such as parks, golf courses, and sports fields [1]. However, 99.7% of the applied load of herbicides is dispersed as residues that enter the aquatic environment through runoff and leaching [2,3]. This can have both direct and indirect negative effects on the aquatic biota and these effects are detectable at multiple levels of biological organization, such as from the molecular to the ecosystem level. There is increasing public awareness of the potential risks that herbicides pose not only to water quality and non-target organisms, but also to human health [4]. Therefore, effective monitoring and management strategies must be developed to maintain the integrity of aquatic ecosystems. Such strategies must be underpinned by accurate quantitative data on both the detection of herbicides in aquatic ecosystems and their risks to aquatic life.

One of the most common herbicides, which is the phenylurea herbicide diuron {3-(3,4-dichlorophenyl)-1,1-dimethylurea)}, has been used in agriculture for over 50 years to inhibit the growth of various annual and perennial weeds, mosses, and agricultural crops [5,6]. Diuron can spread throughout aquatic systems through soil leaching and causes severe environmental pollution and ecological hazards in nearby water bodies [7]. Diuron is a photosystem II (PSII) inhibitor that restricts photosynthetic activity in plants by binding to the D1 protein in thylakoids and strongly blocks re-oxidation of the primary electron acceptor (Q_A_) [8,9]. This PSII inhibitor causes oxidative damage within plant cells via the production of reactive oxygen species (ROS), such as superoxide, hydroxyl radicals, and H_2_O_2_, and reduces CO_2_ fixation and plant growth [10,11,12]. Inhibition of the photosynthetic apparatus by diuron can result in changes in community composition by altering the pigment content (even at low concentrations) and reducing the biomass of important primary producers [13].

The presence of diuron in a variety of environments has been reported worldwide, with concentrations ranging from 0.1–12 ng L^−1^ in seawater to 4,620 ng L^−1^ in freshwater near farmlands and an average of 46.6 × 10^3^ ng L^−1^ in sewage treatment plant sludge [14,15,16]. The half-life of diuron is more than 300 days in groundwater, surface water, and soil as it has relatively stable phenylurea properties [17,18]. In addition, due to the presence of chlorinated groups, diuron has strong toxicity and resistance to biochemical degradation as well as long-term environmental subsistence and high bioaccumulation, resulting in serious harm to human health and the ecosystem [19,20,21]. Due to its high ecotoxicity, the US Environmental Protection Agency and the European Commission have classified diuron as a priority pollutant [22]. Therefore, effective monitoring of diuron residues in aquatic environments is of increasing interest [6]. 

Sophisticated analytical methods such as high-performance liquid chromatography (HPLC) and mass spectrometry are commonly used to measure herbicide residues. However, chemical analysis is a highly specific and sensitive method for herbicide detection, that has several drawbacks including complex sample preparation procedures, the need for expensive chemicals and equipment, and interference from secondary contaminants during analysis [23]. In addition, this purely chemical approach does not provide ecologically meaningful information on temporal changes in exposure or the interactive effects of pollutants [24]. In order to compensate for these limitations, biological assays have been used to assess pollutant-induced ecological risks. In particular, aquatic bioassays are important tools to assess the quality of water containing mixtures and unknown pollutants and to provide safety standards for water management in an ecological context that cannot be expected from conventional chemical analysis-based management since the latter method relies on measurements of single and standardized chemicals. The choice of a model organism for toxicity testing depends on its sensitivity to specific pollutants.

Aquatic macrophytes belonging to the family Lemnaceae, which are commonly known as duckweeds, are attractive experimental model organisms for several reasons; these include their simple structure, homogeneity, ease of culture, and high growth rates [25,26]. They are small vascular plants consisted of floating leaves and submerged roots and they usually grow in stagnant or slow-flowing nutrient-rich water [27,28]. In addition, these plants play an important ecological role as primary producers, are widely distributed in freshwater ecosystems, and are highly sensitive to organic and inorganic substances such as herbicides, pharmaceuticals, and metals [25,29,30]. Therefore, duckweeds have been used for decades in the United States and Europe to assess the effects of a wide range of pollutants [31]. In particular, *Lemna minor* is a preferred test species because it is a model for ecotoxicology, relative metabolomics studies have been conducted, and the toxic potential of biologically active compounds in the aquatic environment has been demonstrated using this species [32,33,34,35]. The International Organization for Standardization (ISO) and the Organization for Economic Co-operation and Development (OECD) have developed a standard growth inhibition test using *L. minor*.

The ultimate goal of bioassay testing is to provide representative and inclusive criteria for exposure conditions, thereby improving risk assessment and water quality management. In this respect, multiple rather than single endpoint testing shows greater potential for more comprehensive risk assessment of toxics. Such an approach provides important insight into the mechanisms underlying toxicity, as well as the relative sensitivity of measured endpoints to toxicant concentration and/or exposure duration, thereby identifying specific endpoints that can effectively detect perturbations by specific phytotoxicants [36]. Many endpoints have been applied to *Lemna*, including frond number, plant number, root number, dry or fresh biomass, frond diameter or area, root length, carbon uptake, chlorophyll content, and so on [30]. 

In the current study, we examined the effects of diuron on *L. minor* using different endpoints at the physiological (growth and photosynthetic efficiency), biochemical (pigment biosynthesis and reactive oxygen species (ROS) levels), and molecular (gene transcription) levels. Our findings provide important insight into the relative sensitivity of different endpoints for diuron toxicity assessment. In addition, they shed light on the toxicity mechanisms of diuron in a model aquatic macrophyte species.

## 2. Materials and Methods

### 2.1. Plant Materials and Culture Conditions

*L. minor* was collected from a shallow pond in Donam-dong, Sangju-si, Korea (36°26′48″ N, 128°15′22″ E). The *Lemna* stock culture was maintained in the laboratory at 25 ± 2 °C under 30–40 μmol photons m^−2^ s^−1^ of continuous light provided by square white LED (Light Emitting Diode) panel lights (340 × 500 × 10 mm; Daewon, Bucheon, Korea). The cultures were maintained in Steinberg growth medium [37] in polypropylene containers (103 × 78.6 mm).

### 2.2. Diuron Toxicity Testing 

The diuron toxicity test was carried out in a controlled environment chamber at 25 ± 2 °C and continuous light of 100 ± 10 μmol photons m^−2^ s^−1^. The test vessels were 6-well plastic plates (well dimension 34.8 mm in diameter, SPL, Seoul, Korea). Each well contained 10.0 mL of test solution and five *Lemna minor* plants, each comprising two fronds (n = 3 plates). Diuron stock solutions were prepared in dimethyl sulfoxide (DMSO; ≥99.9%; CAS No. 67-68-5; Sigma-Aldrich, St. Louis, MO, USA) and were diluted to the desired concentrations (50 × 10^3^ ng L^−1^ and 50 × 10^4^ ng L^−1^) with Steinberg medium. 

Plants were harvested for measurement of the different endpoints after 24, 48, and 72 h of exposure.

#### 2.2.1. Measuring Total Frond Area and Fresh Weight

Following 24, 48, and 72 h of exposure to different concentrations of diuron, the changes in surface area of the plants were measured with an image analyzer. The fronds were then dried on a paper towel and weighed to determine their fresh biomass.

#### 2.2.2. Pigment Contents

Chlorophyll *a* and *b* (Chl *a* and *b*) and carotenoids were extracted from approximately five *L. minor* plants in 1 mL methanol (CH_3_OH; ≥99.9%; CAS No. 67-56-1; Sigma-Aldrich, Co., St. Louis, MO, USA) for 24 h in the dark at 4 °C. The absorption of the supernatant of the methanolic extract was measured using a Scinco S-3100 PDA UV-Vis spectrophotometer at 666 nm (Chl *a*), 653 nm (Chl *b*), and 470 nm (carotenoids). Chl *a*, Chl *b*, and carotenoid contents were estimated using the equations described in Lichtenthaler [38]

#### 2.2.3. Chlorophyll a Fluorescence

Chl *a* fluorescence was measured using a pulse amplitude modulated imaging fluorometer (I-PAM, Walz, Effeltrich, Germany). After 24, 48, and 72 of exposure, the samples were dark-adapted for 15 min and exposed to LED light pulses (0.15 µmol photons m^−2^ s^−1^) to determine the initial fluorescence yield (*F_o_*). The maximum fluorescence yield (*F_m_*) was measured by applying a saturation pulse of approximately 5000 µmol photons m^−2^ s^−1^ emitted from the built-in halogen lamp. The maximum PSII quantum yield (*F_v_*/*F_m_*) was calculated using the following equation.

*F_v_*/*F_m_* = (*F_m_* − *F_o_*)/*F_m_* (1)

Rapid light curves (RLCs) were derived using 10 s pulses of actinic light that increased stepwise from 0 to 1517 μmol photons m^−2^ s^−1^ [39]. The maximum electron transport rate (ETR_max_) was derived from the hyperbolic tangent equation adapted from Platt et al. [40]:

ETR_max_ = (1 *−* exp (*−*α × I/P_t_)) × exp (−β × I/P_t_) (2)

where α represents the rate of electron transport under light-constrained conditions, P_t_ is a theoretical parameter and β is an inhibition coefficient.

Non-photochemical quenching (NPQ) was quantified following Bilger and Björkman in the following equation [41].

NPQ = (*F_m_ − F_m_′*)/*F_m_′* (3)

where *F_m_′* represents the maximum fluorescence yield of a light acclimated state.

#### 2.2.4. Reactive Oxygen Species (ROS) Measurements

ROS production was measured using DHR123 (dihydrorhodamine; Life Technologies, Carlsbad, CA, USA), which is oxidized to a fluorescent compound (rhodamine 123) upon reaction with ROS [42,43]. Briefly, supernatants from *L. minor* homogenates containing 1 mM phosphate-buffered saline (PBS; pH 8) were incubated with 30 μM DHR-123 for 20 min at room temperature. Fluorescence was measured at an excitation wavelength of 485 nm and an emission wavelength of 535 nm. ROS production in each sample was quantified based on the amount of DNA measured by GelGreen (Biotium, Fremont, CA, USA) nucleic acid staining.

#### 2.2.5. RNA Extraction, cDNA Synthesis, and RT-PCR Analysis

Whole plants with fronds and roots were harvested, ground in liquid nitrogen, and total RNA was extracted from the samples using a RNeasy Plant Mini Kit (Qiagen, Hilden, Germany) as per the manufacturer’s protocol. RNA concentrations were determined using a Nanodrop UV spectrophotometry (Thermo Fisher Scientific, Waltham, MA, USA). cDNA was generated from 1 μg of total RNA from *L. minor* using a Diastar RT Kit (SolGent Co., Ltd, Daejeon, Korea).

Primers for quantitative reverse-transcription PCR (qRT-PCR) are summarized in Table 1. qRT-PCR was performed using a CFX Connect Real-Time PCR Detection System (Bio-Rad, Hercules, CA, US). Each 10 μL reaction mixture contained 5 μL of 2X RT PCR Smart Mix (with SYBR Green) (SolGent Co., Ltd, Daejeon, Korea), 10 nM of each primer, and 1 μL of diluted first-strand cDNA. The cycling conditions were as follows: 95 °C for 5 min followed by 40 cycles of 94 °C for 30 s, 57 °C for 30 s, and 70 °C for 10 s in 96-well optical reaction plates. Cycle threshold (C_T_) values were determined for three biological replicates, with three technical replicates for each value. The expression levels of the reference gene (*18S rRNA*) and target gene (*rbcL*) tested were determined based on the Ct values and were calculated using the 2^−^^△△CT^ method [44].

### 2.3. Statistical Analysis

Data were analyzed by one-way analysis of variance (ANOVA) and post-hoc comparisons were performed via the least significant difference (LSD) test to determine differences among treatments. Correlation analysis between 10 endpoints and diuron concentration was performed using the R package ggplot2 (R version 4.0.5). For all tests, *p* < 0.05 was considered statistically significant.

## 3. Results and Discussion

### 3.1. Total Frond Area and Fresh Weight

The most commonly measured endpoint in *Lemna* tests is the frond area. Therefore, we compared the sensitivity of frond area to diuron with that of other endpoints. Total frond area and fresh weight of *L. minor* decreased significantly (*p* < 0.05) as a function of exposure time and concentration of diuron. Diuron has been reported to inhibit plant growth by interfering with electron flow in photosystem II during photosynthesis, resulting in reduced carbon uptake and cessation of carbohydrate production [46]. Both the total frond area and fresh weight are considered to be a result of the overall response to the toxicant and, therefore, follow the changes in the biochemical and physiological processes at cellular and tissue levels.

**Figure 1:**
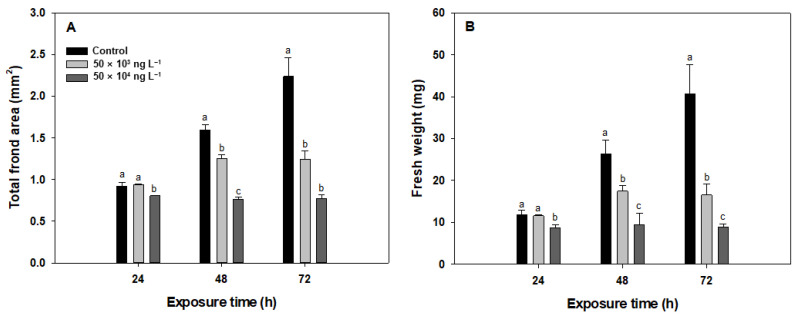
Total frond area (**A**) and fresh weight (**B**) of *Lemna minor* exposed to diuron (50 × 10^3^ and 50 × 10^4^ ng L^−1^) for 24, 48, and 72 h. Data represent mean values of three replicates. Standard deviations are indicated by error bars. Different letters indicate statistically significant differences at *p* < 0.05 (one-way ANOVA, LSD).

As shown in Figure 1, total frond area and fresh weight decreased by approximately 2% compared to the control after 24 h of exposure to both concentrations of diuron. After 48 and 72 h of diuron exposure, the decreases in the total frond area were 20% (159 mm^2^ to 126 mm^2^) and 43% (223 mm^2^ to 124 mm^2^) at 50 × 10^3^ ng L^−1^ and 52% (159 mm^2^ to 76 mm^2^) and 65% (223 mm^2^ to 77 mm^2^) at 50 × 10^4^ ng L^−1^ diuron, respectively (Figure 1A). For fresh weight, 34% (26 mg to 17 mg) and 59% (41 mg to 17 mg) inhibitions were observed at 50 × 10^3^ ng L^−1^ diuron after 48 and 72 h of exposure, respectively, and reductions of 64% (26 mg to 9.0 mg) and 78% (41 mg to 9.0 mg) were observed after 48 and 72 h of exposure, respectively, at 50 × 10^4^ ng L^−1^ (Figure 1B).

### 3.2. Photosynthetic Pigments 

**Figure 2:**
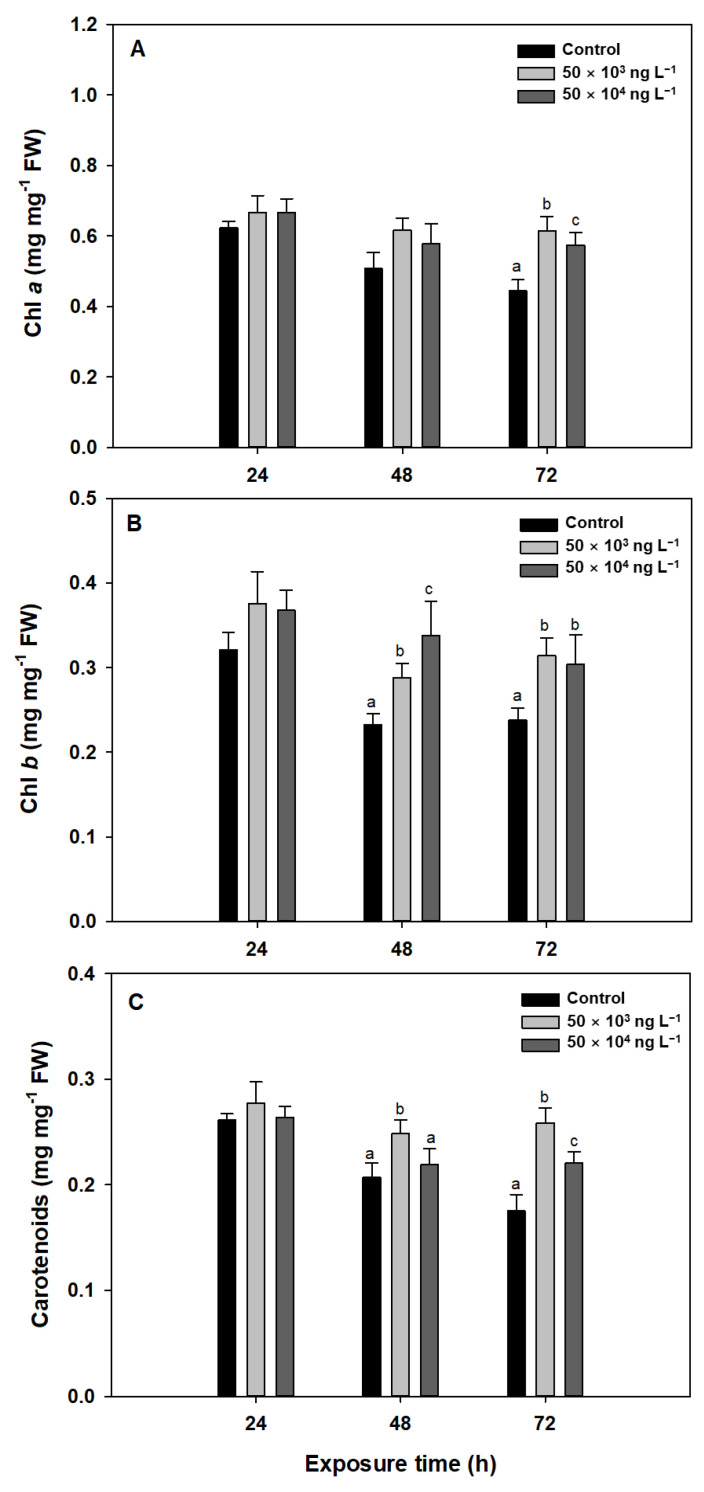
Levels (all in mg mg^−1^ FW) of chlorophyll *a* (**A**), chlorophyll *b* (**B**), and carotenoids (**C**) in *Lemna minor* exposed to diuron (50 × 10^3^ and 50 × 10^4^ ng L^−1^) for 24, 48, and 72 h. Data represent means of three replicates. Standard deviations are indicated by error bars. Different letters indicate statistically significant differences at *p* < 0.05 (one-way ANOVA, LSD). Absence of a letter in a column indicates no statistical difference.

Short-term acute reactions to diuron, as revealed by fluorescent indicators, are potentially reversible, but the diuron can destroy chlorophyll and carotenoids (such as xanthophyll) along with cell membranes upon prolonged exposure which causes irreversible damage [47]. This damage can increase the overall leaf spectral reflectance and the ratio of accessory pigments to chlorophyll [47]. The contents of chlorophylls and carotenoids after exposure to diuron are shown in Figure 2. After 72 h of exposure to 50 × 10^3^ and 50 × 10^4^ ng L^−1^ diuron, Chl *a* content increased significantly by 38% (0.44 mg mg^−1^ FW to 0.61 mg mg^−1^ FW) and 29% (0.44 mg mg^−1^ FW to 0.57 mg mg^−1^ FW), respectively (Figure 2A). However, Chl *a* content was not significantly affected by 24 or 48 h diuron treatment (Figure 2A). The Chl *b* content increased in response to 48 and 72 h of diuron exposure. The amount of Chl *b* was 23% (0.23 mg mg^−1^ FW to 0.29 mg mg^−1^ FW) (48 h exposure) and 32% (0.24 mg mg^−1^ FW to 0.31 mg mg^−1^ FW) (72 h exposure) higher at a diuron concentration of 50 × 10^3^ ng L^−1^ compared to the control (Figure 2B). The carotenoid content also increased significantly after 48 and 72 h of diuron exposure. Even at a diuron concentration of 50 × 10^3^ ng L^−1^, carotenoid levels were increased by 19% (0.21 to 0.25) and 47% (0.18 to 0.26) when compared to levels in the control after 48 and 72 h of exposure, respectively (Figure 2C). 

In plants exposed to diuron-type herbicides, an increase in chlorophyll content was observed, which was attributed to the induction of shade-type chloroplast formation by these herbicides [48]. Shade-type chloroplasts have ultrastructural modifications, such as wider grana and higher thylakoid stacks [49], which may have accommodated more pigments in the chloroplasts. Thus, the reason for the higher chlorophyll content in *L. minor* exposed to diuron than in the control *L. minor* could be due to the formation of shade-type chloroplasts with an accompanying increase in pigment content in diuron-exposed *L. minor*. For carotenoids, it is possible that carotenoids with antioxidant properties were increased in response to ROS production. Macinnis-Ng and Ralph [50] also reported that the total chlorophyll content of seagrass (*Zostera capricorni*) significantly increased after diuron treatment compared to the control group.

### 3.3. Chlorophyll a Fluorescence 

**Figure 3:**
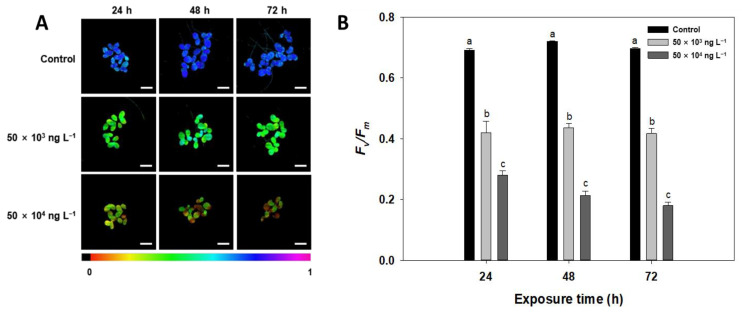
Effects of diuron (50 × 10^3^ and 50 × 10^4^ ng L^−1^) on maximum quantum yield (*F_v_/F_m_*). (**A**) Images of the chlorophyll *a* fluorescence parameter *F_v_/F_m_* in *L. minor* under diuron stress. Chlorophyll fluorescence images were obtained using Imaging PAM (I-PAM, Walz, Effeltrich, Germany). (**B**) The average values of *F_v_/F_m_* in *L. minor*. Values are mean ± standard deviation (n = 3). Different letters indicate statistically significant differences at *p* < 0.05. Scale bar, 5 mm.

In vivo Chl *a* fluorescence is a non-destructive, simple, and rapid quantitative indicator of changes in PSII activity caused directly or indirectly by stress [26,51]. The effect of diuron on PSII photochemical activity has been reported in several studies [10,26,52,53]. Accordingly, derivatives of diuron in water induceed a change in the Chl *a* fluorescence yield of PSII, which is quantitatively correlated with the concentration of this phenylurea herbicide. *F_v_/F_m_* reflects the integrity of the photosystem II/light-harvesting complex II (PSII/LHCII) complex or the intactness of the thylakoid membranes, indicating the intrinsic photochemical efficiency of PSII [54,55]. In the current study, by 24 h diuron exposure had already caused a significant reduction in *F_v_/F_m_* within the tested concentration range, to 39% (0.69 to 0.42) and 59% (0.69 to 0.28) of the control at 50 × 10^3^ and 50 × 10^4^ ng L^−1^, respectively. After 48 h of treatment, the *F_v_/F_m_* values decreased even further (Figure 3A,B). These results suggest that PSII/LHCII or thylakoid membranes are affected by diuron. Kumar and Han [24] also detected a significant decrease in *F_v_/F_m_* with increasing diuron concentration in *L. minor* (*p* < 0.001).

The photosynthetic electron transport rate (ETR) in a plant varies depending on the rate of photon absorption and the efficiency of PSII [56]. Diuron binds to the exchangeable quinine (Q_B_) site of the D1 protein and blocks electron transport beyond the 1-electron reduction in the bound quinone Q_A_, the first stable electron acceptor [24]. In the current study, the maximum electron transport rate (ETR_max_) of *L. minor* exposed to diuron was significantly (*p* < 0.05) suppressed (by greater than ~90%) after 24 h of exposure (Table 2). This reduction in ETR may not only reduce ATP content but also nicotinamide adenine dinucleotide phosphate hydrogen (NADPH) content and plant growth [46]. Park et al. [26] showed that ETR_max_ is the most sensitive endpoint for diuron toxicity in *L. minor*.

Non-photochemical quenching (NPQ) is the fraction of excess energy dissipated as heat by the NPQ photo-protective mechanism [57]. Due to its high sensitivity and rapid response, NPQ is one of the most appropriate indicators of phytotoxicity [58]. Significant reductions in NPQ have been reported in *L. minor*, especially in the presence of 12.5 × 10^3^ ng L^−1^ diuron [24]. Han et al. [59] suggested that a toxicant (i.e., copper (Cu)) may reduce the rate of reactions in the P680-pheophytin-Q_A_-Q_B_ pathway or the electron transport chain. Furthermore, according to Brack and Frank [60], urea herbicides simultaneously reduce photochemical and non-photochemical quenching due to blockages in the electron transport chain. Thus, non-photochemical quenching is also reduced since the proton motive force cannot be built up without electron transport. Therefore, the decrease in NPQ in *L. minor* observed in the current study is likely due to the decrease in the ETR. NPQ values decreased from 0.6 to 0.3 in response to all diuron treatments after 48 and 72 h of exposure (Table 2).

### 3.4. Reactive Oxygen Species (ROS)

**Figure 4:**
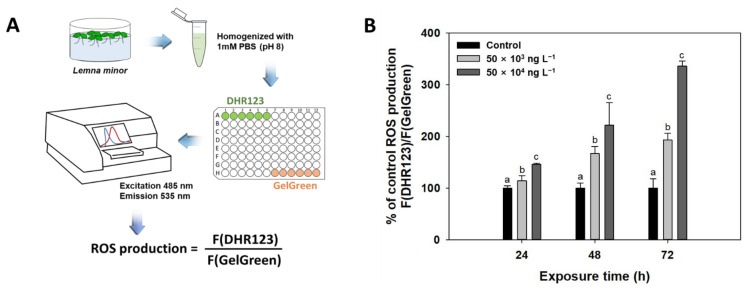
Experimental procedure used to assess ROS levels as DHR123 values normalized to DNA content. Supernatants from *Lemna minor* homogenates containing 1 mM phosphate-buffered saline (PBS; pH 8) were incubated with 30 μM DHR-123 for 20 min at room temperature (**A**). Reactive oxygen species (ROS) production in *L. minor* exposed to increasing concentrations of diuron (0, 50 × 10^3^, 50 × 10^4^ ng L^−1^) for 24, 48, and 72 h (**B**). Bars represent the mean ± standard deviation of three replicates. Different letters indicate statistically significant differences at *p* < 0.05 (one-way ANOVA, LSD).

ROS produced by plants subjected to biological and abiotic stress induce oxidative stress [61]. ROS inhibit chloroplast development in plants, reduce seed survival and root growth, stimulate frond separation and desiccation, and trigger peroxidation of essential membrane lipids in intracellular plasma membranes and organelles [24,62]. Here, we observed a concentration-dependent increase in ROS levels in response to diuron exposure (Figure 4A). As shown in Figure 4B, the ROS concentrations in *L. minor* increased with increasing diuron concentration. Compared to the control, 50 × 10^3^ and 50 × 10^4^ ng L^−1^ diuron increased ROS levels 1.1-fold (100% to 115%) and 1.5-fold (100% to 153%), respectively, after 24 h of exposure; 1.4-fold (100% to 136%) and 2.3-fold (100% to 228%), respectively, after 48 h of exposure; and 2.1-fold (100% to 207%) and 3.6-fold (100% to 363%), respectively, after 72 h of exposure. In addition, diuron causes the depletion of ascorbate, which is a major non-enzymatic antioxidant, and weakens antioxidant enzyme activity in *L. minor* [63].

### 3.5. Gene Transcription

**Figure 5:**
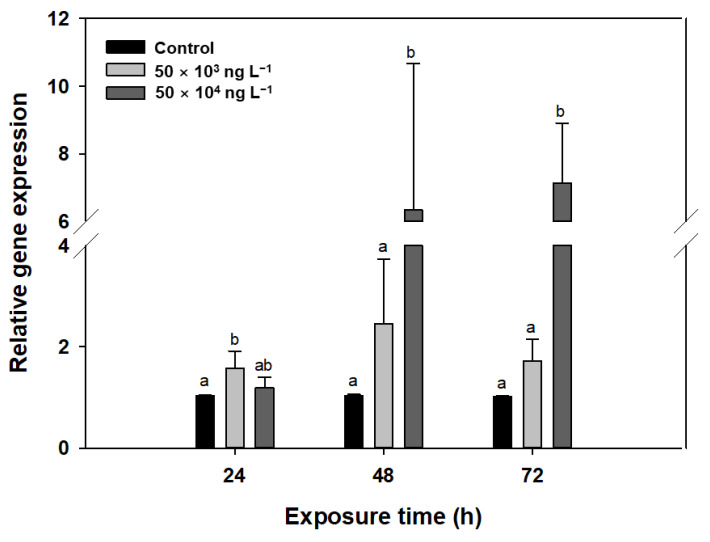
Relative *rbcL* expression in *Lemna minor* under diuron stress. *L. minor* was exposed to different concentrations of diuron (50 × 10^3^ and 50 × 10^4^ ng L^−1^). Data are the means of three replicates (± standard deviation). Different letters indicate statistically significant differences at *p* < 0.05 (one-way ANOVA, LSD).

The transcript levels of *rbcL*, which is the gene encoding Rubisco [64], were significantly higher than those in the control after 48 and 72 h of exposure to diuron, reaching 236% (1.0 to 2.5) and 169% (1.0 to 1.7) of the mean transcript levels in the control following exposure to 50 × 10^3^ ng L^−1^ diuron and 610% (1.0 to 6.3) and 702% (1.0 to 7.1) following exposure to 50 × 10^4^ ng L^−1^ diuron, respectively (Figure 5). Perhaps this upregulation of gene transcription represents the plant’s response to this phytotoxic substance to help maintain photosynthesis and survival. However, this increase may result in more severe oxidative damage via increased ROS production [65]. Further studies are required to support this hypothesis.

The levels of 18S rRNA can be highly dynamic in response to stress; therefore, it seems worthwhile to use a second reference gene from the *L. minor* genome, which will render the analysis much more reliable.

**Figure 6:**
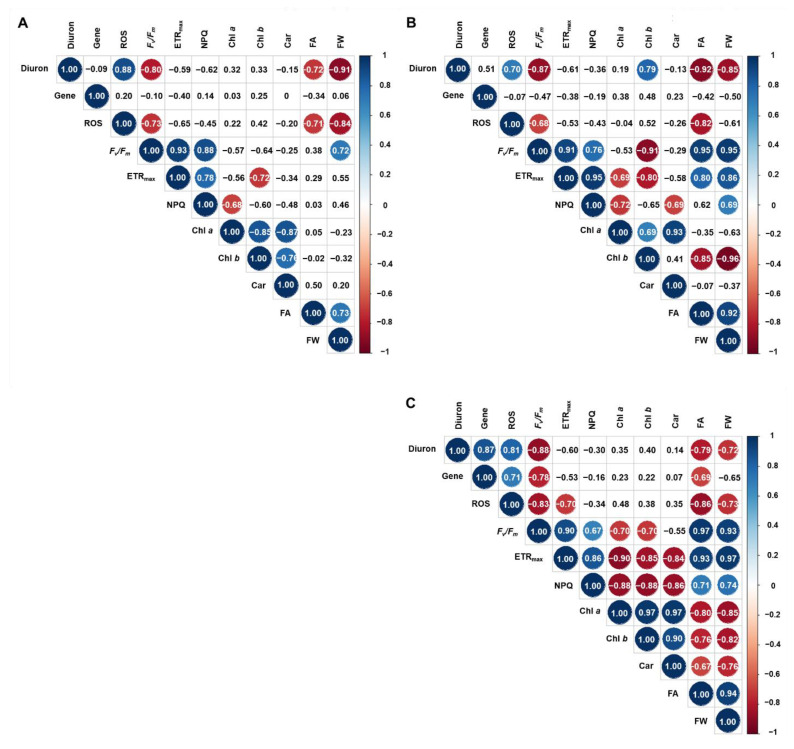
Correlation between 10 endpoints and diuron concentrations in *L. minor* at 24 (**A**), 48 (**B**), and 72 h of exposure (**C**). Only indicators showing statistical significance at the 5% level are shown in circles. The color and size of the circle indicate the strength of the correlation. The color bar on the right indicates the scale of the correlation coefficients. Light blue, blue, and navy blue indicate the correlation coefficients > 0.5, 0.7, and 0.9, respectively, while light pink, orange, and red indicate correlation coefficients < 0.5. The numbers in the boxes are the correlation coefficients. Note that for the sake of clarity, all correlation matrix views show only the upper part of the matrix to avoid duplication. Diuron, diuron concentration; FA, frond area; FW, fresh weight; Chl *a*, chlorophyll *a* content; Chl *b*, chlorophyll *b* content; Car, carotenoid content; *F_v_/F_m_*, maximum quantum yield; ETR_max_, the maximum electron transport rate; NPQ, non-photochemical quenching; ROS, reactive oxygen species production; Gene, relative *rbcL* gene expression.

Figure 6 shows the correlative relationships between 10 endpoints and diuron concentrations (*p* < 0.05). Under diuron stress, pigment, ROS, and gene transcript levels increased while frond area, fresh weight, and photosynthetic endpoints (*F_v_/F_m_* and ETR_max_) gradually decreased over the duration of exposure (Figure 6A–C). Notably, the ROS levels, *F_v_/F_m_*, frond area, and fresh weight were highly correlated with diuron concentration in all the time points examined. The growth endpoints (frond area and fresh weight) showed a strong negative correlation with ROS levels and positive correlations with *F_v_/F_m_* and ETR_max_ (Figure 6B,C). A high correlation was also detected between NPQ and ETR_max_ (Figure 6A–C).

Diuron toxicity was detectable at all endpoints after 48 h of exposure. The order of sensitivity based on the quantitative responses (inhibition or stimulation) of the different endpoints measured after 48 h of exposure to 50 × 10^3^ ng L^−1^ diuron relative to the control was as follows: Gene transcription (136%) > ETR_max_ (95%) > NPQ (51%) > *F_v_/F_m_* (39%) > ROS (36%) > Fresh weight (33%) > Chl *b* (23%) > Chl *a* (21%) > Total frond area (20%) > Carotenoids (19%).

## 4. Conclusions

The current results provide new insight into the mechanism of diuron toxicity in duckweed (*L. minor*) and the sensitivity of different endpoints at the physiological, biochemical, and molecular levels. The most sensitive and least sensitive endpoints in *L. minor* that can be used to assess diuron toxicity are gene transcription (molecular endpoint) and carotenoid content (biochemical endpoint), respectively.

The relatively significant increase in *rbcL* gene transcription in response to 48 h of exposure to diuron represents the plant’s response to this phytotoxic compound even though it may result in more severe oxidative damage due to increased ROS production. This excessive ROS production significantly reduces plant growth since the growth endpoints (frond area and fresh weight) showed a strong negative correlation with ROS levels. In the P680-pheophytin Q_A_-Q_B_ pathway, the ETR can be reduced by xenobiotics such as diuron, which can subsequently interfere with the assembly of PSII and results in decreased NPQ. In this sense, the high correlation between the ETR and NPQ detected in the current study is noteworthy.

## Figures and Tables

**Table 1:** Primers used in this study.

Genes	Sequence
18S Rrna * (housekeeping gene)	Forward: 5′AGAGGAACAGTCGGGGGCATT-3′ Reverse: 5′-CGGCATCGTTTACGGTTGAGA-3′
*rbcL*	Forward: 5′-GTCCATGTACCAGTAGAAGATTCGGC-3′ Reverse: 5′-ATGTCACCACAAACAGAGACTAAAGC-3′

* The sequence cited from [45].

**Table 2:** Effect of diuron (50 × 10^3^ and 50 × 10^4^ ng L^−1^) on induced chlorophyll *a* fluorescence parameters including maximum electron transport rate (ETR_max_) and non-photochemical quenching (NPQ) in *Lemna minor* exposed for 24, 48, and 72 h. Data represent mean ± standard deviation of three replicates. Different letters indicate statistically significant differences at *p* < 0.05 (one-way ANOVA, LSD).

Diuron (ng L^−1^)	24 h Exposure		48 h Exposure		72 h Exposure	
	ETR_max_	NPQ	ETR_max_	NPQ	ETR_max_	NPQ
0	34 ^a^ ± 0.71	0.55 ^a^ ± 0.001	34 ^a^ ± 0.99	0.59 ^a^ ± 0.006	35 ^a^ ± 1.9	0.56 ^a^ ± 0.02
50 × 10^3^	0.75 ^b^ ± 0.09	0.47 ^b^ ± 0.02	1.5 ^b^ ± 0.12	0.28 ^b^ ± 0.004	1.5 ^b^ ± 0.24	0.31 ^b^ ± 0.01
50 × 10^4^	0.12 ^b^ ± 0.09	0.45 ^b^ ± 0.02	0.01 ^b^ ± 0.00	0.36 ^c^ ± 0.01	0.01 ^b^ ± 0.00	0.38 ^b^ ± 0.04

## Data Availability

All the results found are available in this manuscript.
